# Effects of Ornamental Plant Density and Mineral/Plastic Media on the Removal of Domestic Wastewater Pollutants by Home Wetlands Technology

**DOI:** 10.3390/molecules25225273

**Published:** 2020-11-12

**Authors:** Luis Carlos Sandoval-Herazo, Alejandro Alvarado-Lassman, María Cristina López-Méndez, Albino Martínez-Sibaja, Alberto A. Aguilar-Lasserre, Sergio Zamora-Castro, José Luis Marín-Muñiz

**Affiliations:** 1 División de Estudios de Posgrados e Investigación, Tecnológico Nacional de México/Instituto Tecnológico de Orizaba, Oriente 9, Emiliano Zapata Sur, C.P. Orizaba 94320, Veracruz, Mexico; lcsandovalh@gmail.com (L.C.S.-H.); lassman@prodigy.net.mx (A.A.-L.); albino3_mx@yahoo.com (A.M.-S.); albertoaal@hotmail.com (A.A.A.-L.); 2 División de Estudios Posgrado e Investigación, Tecnológico Nacional de México/Instituto Tecnológico Superior de Misantla, Misantla, Veracruz, C.P. Misantla 93821, Veracruz, Mexico; mclopezm@misantla.tecnm.mx; 3 Faculty of Engineering, Construction and Habitat, Universidad Veracruzana Bv. Adolfo Ruíz Cortines 455, Costa Verde, C.P. Boca del Rio 94294, Veracruz, Mexico; szamora@uv.mx; 4 Sustainable Regional Development Academy, El Colegio de Veracruz, Xalapa 91000, Veracruz, Mexico

**Keywords:** wetlands, wastewater, water pollutants, density of plants, phytoremediation

## Abstract

Wastewater treatment (WWT) is a priority around the world; conventional treatments are not widely used in rural areas owing to the high operating and maintenance costs. In Mexico, for instance, only 40% of wastewater is treated. One sustainable option for WWT is through the use of constructed wetlands (CWs) technology, which may remove pollutants using cells filled with porous material and vegetation that works as a natural filter. Knowing the optimal material and density of plants used per square meter in CWs would allow improving their WWT effect. In this study, the effect of material media (plastic/mineral) and plant density on the removal of organic/inorganic pollutants was evaluated. Low (three plants), medium (six plants) and high (nine plants) densities were compared in a surface area of 0.3 m^2^ of ornamental plants (*Alpinia purpurata*, *Canna hybrids* and *Hedychium coronarium*) used in polycultures at the mesocosm level of household wetlands, planted on the two different substrates. Regarding the removal of contaminants, no significant differences were found between substrates (*p* ≥ 0.05), indicating the use of plastic residues (reusable) is an economical option compared to typical mineral materials. However, differences (*p* = 0.001) in removal of pollutants were found between different plant densities. For both substrates, the high density planted CWs were able to remove COD in a range of 86–90%, PO_4_-P 22–33%, NH_4_-N in 84–90%, NO_3_-N 25–28% and NO_2_-N 38–42%. At medium density, removals of 79–81%, 26–32, 80–82%, 24–26%, and 39–41%, were observed, whereas in CWs with low density, the detected removals were 65–68%, 20–26%, 79–80%, 24–26% and 31–40%, respectively. These results revealed that higher COD and ammonia were removed at high plant density than at medium or low densities. Other pollutants were removed similarly in all plant densities (22–42%), indicating the necessity of hybrid CWs to increase the elimination of PO_4_-P, NO_3_-N and NO_2_-N. Moreover, high density favored 10 to 20% more the removal of pollutants than other plant densities. In addition, in cells with high density of plants and smaller planting distance, the development of new plant shoots was limited. Thus, it is suggested that the appropriate distance for this type of polyculture plants should be from 40 to 50 cm in expansion to real-scale systems in order to take advantage of the harvesting of species in these and allow species of greater foliage, favoring its growth and new shoots with the appropriate distance to compensate, in the short time, the removal of nutrients.

## 1. Introduction

Water pollution and low investment in wastewater treatment (WWT) in developing countries results in a high risks for human health and ecosystems [1,2]. This condition creates the obligation to find both economic and ecological alternatives for WWT based on the needs of these regions, located mostly in tropical and inter tropical areas with little attention to such water problem [3,4]. Constructed wetlands (CWs) are a sustainable option to treat wastewater. These engineering systems can mimic the functions of natural wetlands through physical, chemical and biological processes [5], where the role of plants and support media or substrates is essential.

The substrates serve as the support of the living organisms, provide storage for many contaminants and permanent physical support for plants [6,7]. Conventionally, the use of minerals such as tezontle, sand, zeolite and gravel are very common in CWs as a substrate or filling material [8,9]. However, these are usually the most expensive materials during the construction of CWs owing to their commercial use in the construction industry and this is a limitation in rural communities with limited economic resources [10].

In this sense, materials with the same or better functionality and lower commercial costs such as the polyethylene terephthalate (PET) are necessary. Despite the fact that PET causes major negative environmental effects, the roughness and porosity of certain parts of a PET bottle such as the cap, neck and bottom can be functional as a substrate in CWs [11]. Another important component in CWs is the vegetation, commonly called macrophytes in natural wetlands, that have been used in CW systems (*Phragmites australis*, a species of the genus *Typha* spp., *Scirpus* spp. and *C. papyrus*), which provide oxygen to the systems in the substrates, favoring the growth of aerobic bacterial communities. They also contribute to the removal of pollutants by absorption (15 to 25%) of residual water elements used for their development [12,13]. In this last decade, new ornamental flowering plants (OFP, eg., *Anthurium* spp., *Canna* spp., *Heliconiaceae* spp., *Spathiphyllum wallisii*, and *Zantedeschia aethiopica*), not typical of natural wetlands that favor aesthetic aspects of the CWs without affecting the removal function (up to 80% of organic and inorganic compounds), have been evaluated to facilitate the adoption of this technology in small communities [14,15]. The majority of studies regarding CWs have been focused on effect of natural wetland macrophytes, different mineral substrates or water flow type on pollutant removal [12,16,17]. However, little is known about the effects of OFP and the density (number of plants) on the biogeochemical role in WWT. Similarly, little has been studied about the reuse of plastic substrate as filter media. The objective of this study was to compare the removal effect on organic and inorganic pollutants of domestic wastewater of the plant density (three OFP species, one of each one, three OFPs, with two of each species and six OFPs, using two of each species), the use of mineral substrate (porous rock river –PRR) and the reutilization of PET residues as filter media. In this regard, two hypotheses were put forth: (1) High plant density would provide more oxygen to the substrate increasing the aerobic conditions and therefore the pollutant removal efficiency. (2) Knowing the roughness and structure of certain components of PET bottles, such residues can have a similar action as filter media compared with mineral substrates.

## 2. Results and Discussion

### 2.1. Control Parameters

Table 1 shows results of the control parameters monitored during the study period. The water temperature after passing through the systems was reduced by 2 °C on average. In spite of this, it was found to be in a suitable range (between 16.5 and 32 °C) to favor the processes of removal of contaminants in the CWs. On the side of increased bacterial activity, which is initiated at a temperature of >20 °C [18] the results of this study are in these ranges (Table 1). As for the appropriate pH in CWs to promote most of the biological reactions, this occurs in ranges from 6 to 8 [19]. In this study, an average of 6.8 (pH) was detected for the influent, whereas in the effluent the values increased statistically (*p* < 0.05, Table 1). In systems with vegetation, the average pH was 7.7, while in systems without vegetation it was 7.8. These ranges are suitable for promoting biological reactions in CWs. In addition to the above, the low presence of DO in the systems (Table 1), could have favored the increase in pH [20]. In relation to the electrical conductivity (EC), 1601.3 ± 291.2 was observed in the influent and was significantly reduced (*p* > 0.05) in both systems with vegetation (30–36%) and without vegetation (34%) (Table 1). In the literature, it is reported that for a hydraulic retention time less than 3 days, this behavior is not frequent [21]. These results could be due to the capture of micro and macro elements of ions by plants and eliminated through radical absorption and sedimentation of suspended particles [22]. 

The dissolved oxygen (DO), shown in Table 1, increases considerably in systems with vegetation (*p* > 0.05), while in systems without vegetation the increase was not significant (*p* > 0.05) in relation to the influent. The presence of DO indicates the type of conditions found in the systems: aerobic, anaerobic/anoxic for biological processes [23]. Therefore, the presence of oxygen in systems with greater vegetation than in non-planted systems could be due to its release in the radical zone [24,25]. In relation to the total dissolved solids (TDS), these mainly include organic salts and residues, therefore, in terms of their decrease in treatment systems it becomes an indicator of their effectiveness [26]. In this study, a significant decrease of TDS was found (*p* > 0.05) in relation to the value of the influent (Table 1), these values found, after treatment in CWs, are within the limits allowed by the United States Environmental Protection Agency [27] which indicates that 500 mg L^−1^ is a suitable range for discharges to surface bodies and are consistent with those reported by other studies in similar conditions using CWs, where reduced values have been found after treatment in these systems [28].

**Figure 1:**
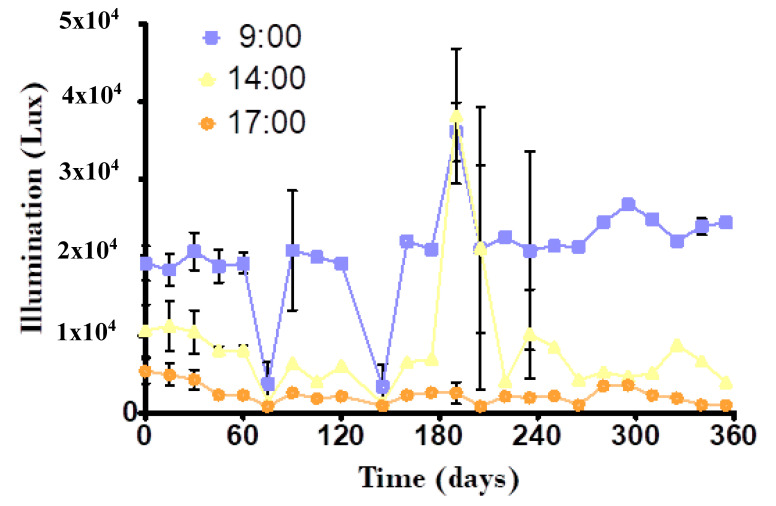
Intensity of light in three different times of day (9, 14 and 17 h) during the study.

An environmental characteristic, such as the intensity of light, played an important role in the development of vegetation. In this study, the range of light oscillated between 2000–5000 lux, 3000–10,000 lux, and 3000–35,000 lux at 17:00, 14:00 and 9:00 h, they favored the development of plants without inhibiting their growth or physiological health. In the same way, such light values favored the observed temperatures for good biological functioning in the system (Figure 1).

### 2.2. Plant Growth and Flower Production

#### 2.2.1. *Alpina purpurata*

**Figure 2:**
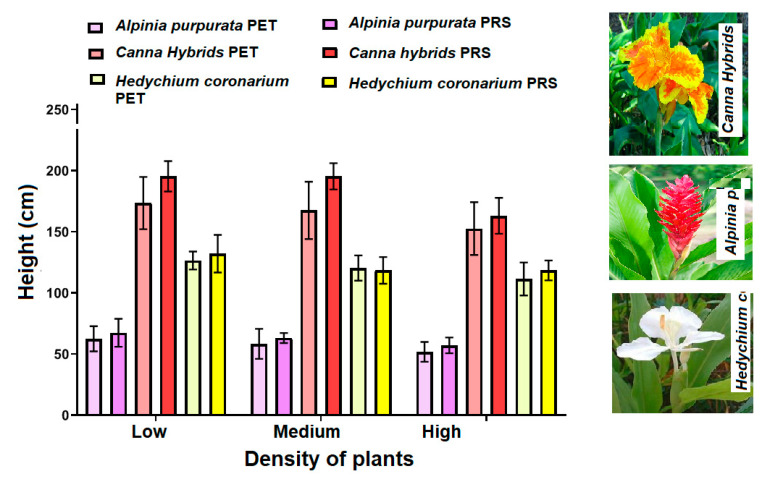
Average maximum height of the vegetation under study in the three different plant densities (under PET and PRS substrates).

*Alpina purpurata* is an ornamental plant listed as an exotic tropical plant in the global weed compendium [29]. It has been cultivated in tropical areas, being capable of surviving in totally or partially flooded soils [30]. Owing to this, it was used as an emerging plant in subsurface horizontal flow CWs. The plant development data of *A. purpurata* are shown in Figure 2. No significant differences (*p* > 0.05) were found in the development of the species in the two-culture media (PET and PRS). The tropical conditions where the study was developed favored its adaptation in CWs indicating that the environmental conditions were adequate for the development of this species [31]. During the study, it was found that plants planted in densities of one individual in polycultures of three plants (low density), by cell, plants produced on average 10 shoots of *A. pulpurata* in both substrates during the 12 months of study, while in a system with polycultures of medium density (three plants), for *A. pulpurata*, for each CW, only six shoots were generated on average, independently of the material support (PET/PRS). In polycultures of nine individuals (high density) *A. purpurata*, produced four shoots per plant. This could be due to the presence of a greater number of plants in the same area (0.36 m^2^), given that in the systems it was possible to generate more competition for the nutrients present in wastewater and hindering the growth of tillers. It should be noted that these species are not commonly used in CWs. In this sense, these data are the first reports of their development in this type of system, finding that the distance of planted and the volume of seedlings significantly influenced their development. As for the adequate distance to improve their development in this study, it was 50 cm and in low density conditions. In relation to the production of flowers, no flowers were produced during the study, which could be due to the fact that, during the flowering period, this plant under terrestrial conditions grows from 1.5 to 2 m tall in an approximate period of 8 months [32]. However, in this study very small plants (10 to 25 cm) were planted and they did not show the same growth behavior as in their natural state, given the new conditions to which they were exposed for development. Thus, studies using a longer evaluation period are necessary to know the rate of flowering of these species in CWs and encourage their ornamental use in future designs.

#### 2.2.2. *Hedychium coronarium*

Tropical floriculture is in early stages in Mexico, although the cultivation of *Hedychium coronarium* shows great potential in various humid tropical areas of the country. This is an herbaceous plant that develops at temperatures above 10 °C [33]. The ambient temperature conditions that occurred in the study area (20–26 °C), as well as the light intensity (Figure 1), showed ranges that could favor the proper development of *H. coronarium*; these can reach up to 3 m high in their natural state. In this study, the PET and PRS substrates were used as media using different planting distances for this plant (Table 1). These differ greatly from those recommended for cultivation (1 to 1.5 m distance between them), which could indicate why the plants did not achieve heights they should have presented (2 to 2.5 m high) in 12 months of study.

Figure 2 shows that regardless of the substrate, on average the plants grew up to 1.5 m high without significant differences between substrates (*p* = 0.621), but failed to produce flowers, although their typical flowering period is from 7 to 8 months after planting [34]. This could be due to the conditions of constant saturation to which they were exposed, such as vegetation saturation, water quality or the reduced space where they were planted. Consequently, new studies are required where this plant is used as emergent vegetation in large-scale systems that allow its better development, more distances for planting and with longer study periods in order to evaluate its flower production in CWs.

#### 2.2.3. *Canna hybrids*

*Canna hybrids* is a tropical plant that is easy to grow and does not die easily because it is resistant to adverse conditions, and even when irrigated with highly polluted water, they reproduce quickly and generally flower 1 year after having been planted. It is considered an invasive plant when it grows wild among controlled crops [35]. In this study, light ranges (Figure 1) were found to be lower than those reported as optimal for cultivation of this OFP, which are between 40,000 and 60,000 lux [36], however, this did not hinder their development.

Significant differences (*p* = 0.001) were found between the vegetation planted in PET and PRS substrates (Figure 2). The plants presented an average of 30 cm more in height in PRS substrate than in PET, although the growth is among the averages reported on the growth of plants (1 to 3 m) [37]. All plants grew healthy and managed to produce on average 95 flowers per plant in PET and 98 in PRS; without finding significant differences in flower production between substrates (*p* = 1.00). Although the use of PET as a substrate in CWs is not common, the plants managed to adapt and grow healthily as in any other common medium such as PRS. According to the growth conditions that are shown in their natural state, these plants generate about 60 flowers per plant in 12 months [38]. However, in this study the flowering increased up to 40%, compared to when it is cultivated in soil, which could be due to the availability of constant nutrients present in wastewater.

### 2.3. Contaminants Concentration in Influents and Effluents from CWs

**Figure 3:**
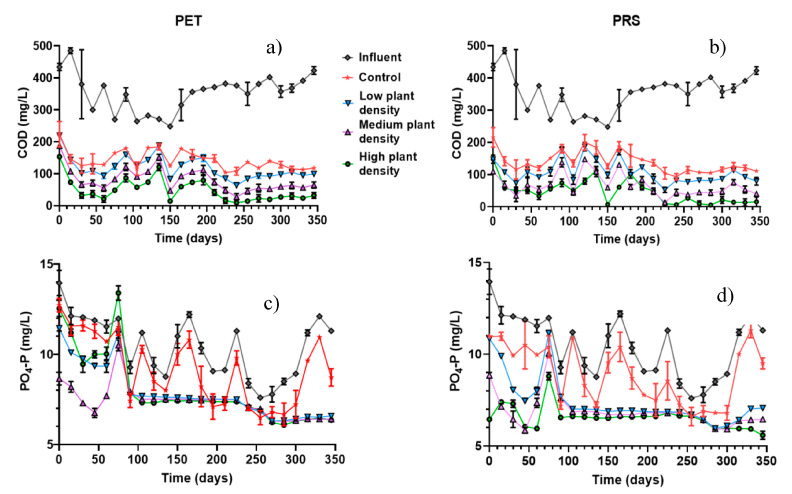
Contaminant concentration, (**a**) COD in PET, (**b**) COD in PRS, (**c**) PO_4_-P in PET and (**d**) PO_4_-P in PRS in effluents and influents in CWs with PET and PRS substrates.

The chemical oxygen demand (COD) is the amount of oxygen needed to oxidize organic carbon completely to CO_2_, H_2_O and ammonium. COD does not differentiate between biologically oxidizable organic matter and biologically inert materials [26]. The COD influent concentrations on average were 353.96 ± 9.21 mg/L. After treatment with CWs, in both substrates it was found that in systems with vegetation and PET the COD was 78.57 ± 2.64 mg/L, and 76.23 ± 6.98 mg/L with PRS (Figure 3b). CWs without vegetation had averages of concentrations of 142.55 ± 1.93 mg/L and 138.54 ± 5.29 mg/L in PET and PRS substrates, respectively (Figure 3a,b). The COD data, after treatment, fulfilled the regulations of the European Union for protection of water quality for discharge into lakes (COD; <125 mg/L). These results may be due to the greater presence of oxygen (Table 1) in systems with vegetation where the greatest COD reductions in CWs were presented after treatment [39]. The COD parameters indicate the ability to reduce oxygen present in receiving bodies of water and are the main parameters for measuring the content of organic matter in wastewater [40,41]. On the other hand, the COD indicates the presence of non-municipal origin substances in the water [42]. 

**Figure 4:**
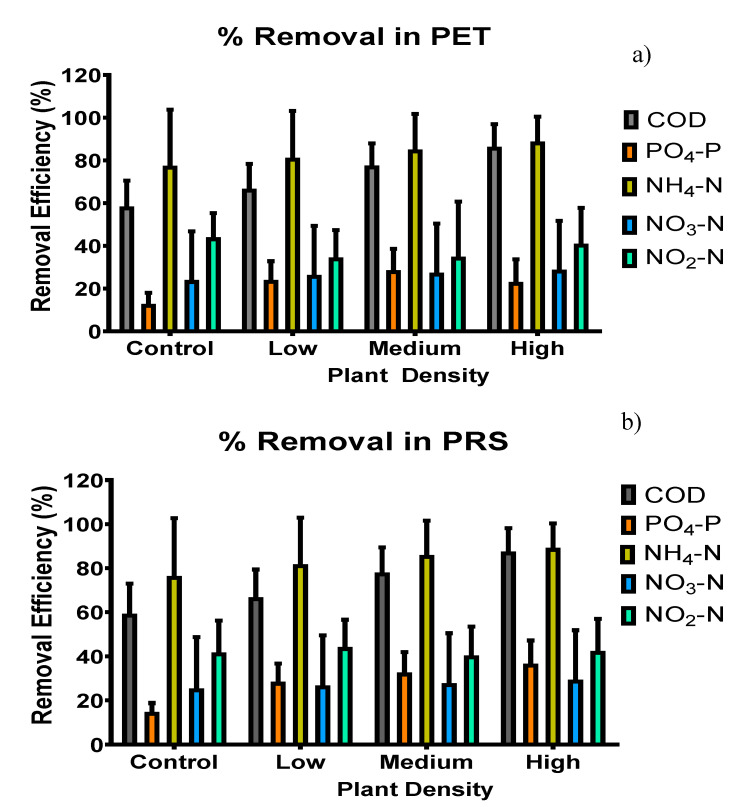
CWs contaminants: (**a**) Removal in PET and (**b**) Removal in PRS.

In this sense, it is essential that they be removed before discharging wastewater into rivers. In this study, systems without vegetation showed removal efficiencies between 58 and 59% in both substrates (Figure 4a,b). In general, the average of removal found in CWs with vegetation was 81% and consistent with the reported by [5,14] with 78%, which is the average of removals found in systems with polyculture of ornamental plants in CWs fed with domestic wastewater. However, significant differences were found according to plant densities (*p* = 0.001) in both substrates and no significant differences were observed regarding the substrate (*p* = 0.07), but different plant density showed significantly higher average of COD removal (*p* = 0.001). CWs with low plant density (65–68%) < medium density (79–81%) < high plant density (86–90%) (Figure 4a,b). This behavior could be due to the release of radical oxygen given the higher plant density in the systems. This information is consistent with that reported by [43], which indicated that the higher the plant density, the better the COD removal in horizontal flow CWs. These results show a clearer picture of the role of vegetation in CWs. However, new studies with a greater presence of organic matter in wastewater are suggested to deal with CWs in high plant polyculture density.

The phosphate concentration (PO_4_-P) in the influent was 10.46 ± 0.25 mg/L. This value decreased significantly (*p* = 0.001) in CWs with vegetation 7.74 ± 0.11mg/L and 6.69 ± 0.15 mg/L in PET and PRS respectively (Figure 3c,d), compared to the systems without plants (9.18 ± 012 mg/L in PET and 8.92 ± 0.25 mg/L in PRS), which could be due to the absorption of PO_4_-P ion by plants for growing development [44], as shown in Figure 2. This behavior is explainable given the main phosphorus removal mechanisms in wetlands (by substrate adsorption and assimilation by plants [45]. However, these values do not comply with the Mexican standard which specifies phosphorus as total phosphorus (PT) and establishes a maximum allowable limit of 5 mg/L. In biologically treated domestic wastewater, PO_4_-P is approximately 80% of PT. Therefore, it is recommended to evaluate these substrates in conjunction with others that have a greater ionic capacity to attract the phosphorus present in domestic wastewater with phosphate content greater than or equal to the values present in this study.

In the PET substrate, the removal in systems without the presence of vegetation was on average 10%, 20% in CWs with low density, 26% with medium density where the highest removal of PO_4_-P occurred; *p* = 0.001) and 22% of removal in CWs with high density [46], indicating that phosphorus in its different forms can be used by plants for their development with better results in tropical climates, where the plants are harvested continuously so that their development is not interrupted until the plants reach maturity. That might explain the results obtained in this study, where the plants managed to develop considerably (Figure 2) and the phosphate could have been used for its development and flowering [18]. The ionic interaction with the substrates was not as relevant in this study, perhaps because of the substrates used (PET and PRS), since using them in CWs is unconventional and precise mechanisms of their functioning are unknown. The elimination in these could be due to the adoption in the microbial films that were developed in the PET substrate. Regarding the removals in PRS substrate, significant differences were found (*p* = 0.001), among all treatments in controls without vegetation 20% <in treatments with three species of plants 26% <in treatment with six individuals of plants 32% <in treatment of nine individuals of 33% plants (Figure 4a,b).

In comparison to the treatments in PET and PRS, the phosphate removal behavior in PET substrate was as expected, given that it apparently did not have an effect on the removal of this compound, while in the PRS treatments a positive effect was shown, but not greater than those reported in the consulted literature [47].

**Figure 5:**
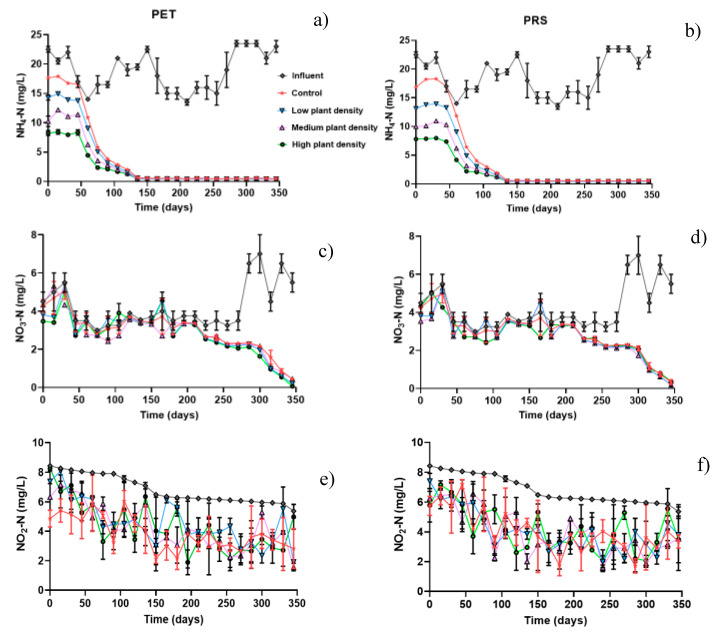
Concentrations of the contaminants, (**a**) NH_4_-N in PET, (**b**) NH_4_-N in PRS, (**c**) NO_3_-N in PET, (**d**) NO_3_-N in PRS, (**e**) NO_2_-N in PET, and (**f**) NO_2_-N in PRS substrates.

Ammonium is removed in CWs, through the action of aerobic and anaerobic microorganisms, taking into account that under anaerobic conditions the reductions are slow [48], which may explain its behavior in this study. The concentrations of ammonium (Figure 5a,b) in the influent oscillated in a ranged of 18.88 ± 0.51 mg/L; these were significantly reduced (*P* = 0.001) in presence of plants and in the systems without plant species. For both substrates from the first 50 days, the removal in the CWs began to be observed, the elimination of pollutants were almost at 100% on the 125 days of study. On average 2.81 ± 0.24 mg/L of NH_4_-N were observed in systems with plants for both substrates, while in units without vegetation 3.23 ± 0.14 mg/L were detected, these results were lower than the limit established by the Mexican law about ion discharge in rivers (NMX-AA-026-SCIF-2001, 25 to 50 mg/L).

The ability of horizontal flow CWs to oxidize ammonia nitrogen is low (nitrification), given the low availability of aerobic zones within the wetland (only in the rhizosphere zone) (Table 1) [49]. Despite this, the eliminations in this study (Figure 4a,b) were highly significant (*p* = 0.0012): in a system with three plants the removal on average was 79 and 80%; in systems with six plants 80 and 82%; in systems with nine plants 61 and 64%, and in controls without vegetation 84 and 90% in PET and PRS substrate respectively. There were no significant differences (*p* = 0.086) between substrates, in contrast with (*p* = 0.094) plant density, as well as between controls. These results are higher than those reported by [50], who used plastic rings as substrates; a combination of zeolite and fly ash with more calcium element; and fly ash using microphytes as emergent vegetation, finding eliminations between 40 and 58% of N-NH_4_.

For nitrate removal, it is important to describe that nitrogen removal from water and wastewater involve a combination of aerobic nitrification and anaerobic denitrification, a significant part of organic nitrogen is converted to ammonia, which is oxidized in aerobic condition by *Nitrosomonas* and *Nitrobacter*. Nitrification implies a chemolithoautotrophic oxidation of ammonia to nitrate under strict aerobic conditions. In HSSF-CWs the aerobic conditions occur mainly in the rhizosphere zone. [51,52]. The concentration of NO_3_-N (Figure 5c,d) in the influent was 4.22 ± 0.18 mg/L for both substrates, and after treatment it decreased significantly (*P* = 0.001) in systems with vegetation, 2.97 ± 0.10 mg/L in PET and 2.91 ± 0.18 in PRS respectively were detected, but not in systems without vegetation (3.65 ± 0.83 mg/L and 2.81 ± 0.18 mg/L in PET and PRS substrate).

Another way of reducing NO_3_-N is through denitrification. NO_3_- is reduced in anoxic conditions by heterotrophic bacteria (such as *Pseudomonas*, *Micrococcus*, *Achromobactor* and *Bacillus*), in this process, denitrifying bacteria decreases inorganic nitrogen such as nitrate and nitrite into innocuous fundamental nitrogen gas [48,52]. In HSSF-CWs, anoxic conditions occur where there is no contact with the vegetation roots.

CWs filled with PET and with presence of vegetation ranged from 24–30% (Figure 4a) and showed significantly higher average nitrate removal efficiency than systems without plants (21%). No significant differences were observed for such ion respect to plant densities (*p* ≥ 0.05), while in CWs with PRS substrate (Figure 4b) the nitrate removal was similar in all the units, regardless of the plant density (25–30%). The results found in this study differ from those expected in horizontal flow CWs for the elimination of N-NO_3_, since anaerobic conditions, a carbon source and denitrifying bacteria favor the elimination of this compound [53]. The previously described since the concentrations of ammonium (Figure 5a,b) were relatively higher, nitrates were produced that could affect the elimination mechanisms of this CWs.

The concentration of NO_2_-N in the influent was 6.84 ± 0.14 mg/L (Figure 5e,f), while in the influents with vegetation was 4.38 ± 0.13 mg/L in PET and 3.99 ± 0.24 mg/L in PRS, in CWs without vegetation it was 3.87 ± 0.10 mg/L and 4.04 ± 0.25 mg/L in PET and PRS respectively (Figure 5e,f). The NO_2_-N elimination was favored in CWs (Figure 4a,b), finding significant differences (*p* = 0.001) between substrates and plant density in cells with controls without vegetation 41 and 30%; in cells with three plants 31 and 40%; in cells with six plants 39 and 41%, and in cells with nine plants 38 and 42% in PET and PRS substrates, respectively. These results may be due to the high removal of ammonium that could contribute to the production of nitrites as shown in Figure 4.

## 3. Materials and Methods

### 3.1. Study Area

This study was carried out in Pastorias, Actopan (June 2016–May 2017). This community is in the central mountainous area of the State of Veracruz, Mexico (−96°57′08″ and 19°55′83″ S). The climate in the area is classified as semi-warm humid with rains throughout the year (45%), warm-humid with rains throughout the year (38%) and warm-humid with abundant rains in summer (17%), with higher temperatures in June and lower in January, at an altitude of 1900 m above sea level. The average annual rainfall is 947.1 mm and the average annual temperature is between 20–26 °C, which are typical conditions of the subtropical areas of America [54].

### 3.2. Constructed Wetlands (CWs) Systems’ Characteristics

**Figure 6:**
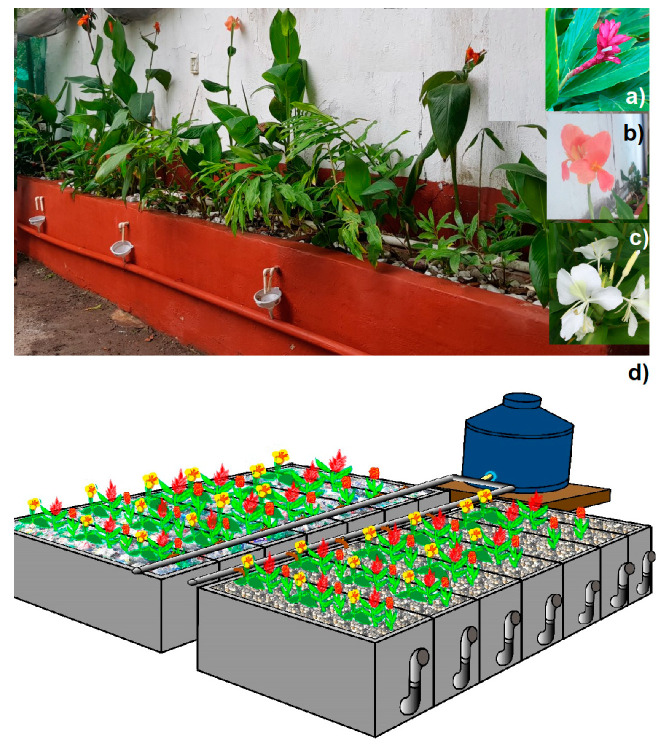
Constructed wetland systems. (**a**) *Alpinia purpurata*, (**b**) *Canna hybrids*, (**c**) *Hedychium coronarium*, (**d**) Scheme of CWs in study.

The CW systems were Horizontal Subsurface Flow (HSSF) cells. Fourteen mesocosms constructed with cement and blocks were used as experimental units (0.70 m. high, 1.5 m. long and 0.24 m. wide; Figure 6). They were installed in a rural dwelling of three people and employed as a WWT home. The water before entering the mesocosms had a pretreatment in a filter settler, in a 1500 L tank. All the 14 experimental units (Figure 6) were filled from bottom to a height of 10 cm with PRR of 8 to 15 cm in diameter. Then, seven mesocosms were filled with 60 cm of PRR as a substrate (2.5 to 3.5 cm diameter, and an uneven surface with 0.4 average porosity), taken from the local Topiltepec river. The other seven cells were filled with 50 cm of PET residues (rough sections were taken and with folds of recycled bottles used for beverages like water and soft drinks, with 2.5 to 3.5 cm in diameter, in order to provide a favorable surface for the development of bacterial communities, with a 0.5% porosity). For cells with PET, 10 cm of PPR were added under the PET to prevent plastic buoyancy; this last fraction (10 cm) did not interfere with the treatment system.

Two filled experimental units, one from PRR and one from PET functioned as controls without vegetation. In the other twelve cells, six were filled with PRR and six filled with PET. The configuration of vegetation density for each different porous media was (15 to 20 cm high each plant): three plants with one sample of *Alpinia purpurata* + one sample of *Canna hybrids* + one sample of *Hedychium coronarium* (low density, 0.4 m distance between plants). Two mesocosms were planted with OFPs consisting of two *A. purpurata* + two *C. hybrids* + two *H. coronarium* plants (medium density, 0.2 m of distance between plants). The other two mesocosms of each filter media were planted with OFPs that included three *A. purpurata* + three *C. hybrids* + three *H. coronarium* plants (high density, 0.1 m of distance between plants). The ornamental plants were collected from areas near the study sites to favor their adaptation to new flood conditions to which they were exposed. All mesocosms were operated with a hydraulic retention time of 4 days.

### 3.3. Sampling and Analysis

From the day the tank was fed with 100% residual water and during the study period, a sample of the influent and effluent of each mesocosm was taken every 15 days. The pollutants analyzed were COD, PO_4_-P, NH_4_-N, NO_3_-N and NO_2_-N in duplicates by standard methods [55]. Total dissolved solids (TDS), electrical conductivity (EC), pH, dissolved oxygen (DO) and water temperature were measured with a H198194 multiparameter meter (Hanna, Ciudad de México, Mexico) placed in the influent and effluent streams of the mesocosms. In addition to these data, the ambient temperature and light intensity were measured every 15 days with a HTC-1 hydrometer (Uplayteck, Ciudad de México, Mexico) and a HIELEC-MS8233-2000 (STEREN, Veracruz, Mexico) lux meter, respectively, at times of 9:00, 14:00 and 17:00 h; estimating and recording the average of each measurement. The height of the plants was measured with a tape measure and the number of flowers was recorded every 30 days.

### 3.4. Statistic Analysis

The response variables were COD, PO_4_-P, NH_4_-N, NO_3_-N, and NO_2_-N, as well as the height of the plant and the number of flowers. Statistical differences between treatments were estimated using a two-way ANOVA with plant density and substrates as factors, followed by evidence of least significant differences (LSD), with a significance level of 5%. All statistical analyses were performed with version 16.1.0 of Minitab (ver. 16, State college, PA, USA).

## 4. Conclusions

The use of *Alpinia purpurata*, *Canna hybrids* and *Hedychium coronarium* in polycultures for treatment of domestic wastewater in mesocosms of CWs favored the elimination of organic matter and nitrogen compounds, while phosphate ion was less removed by such species during the 350 days of study. Owing to this, their use in CW designs is recommended. In addition, *Canna hybrids* flowers showed greater flowering compared to their normal development in the natural state. However, the species *Alpinia purpurata* and *Hedychium coronarium* failed to produce flowers. More studies over a longer period are suggested in order to understand the flowering behavior of these plants in CWs. Apparently, in these systems this may require periods longer than 12 months. Regarding plastic materials, PET was proven to be a suitable support medium for use in large-scale CWs, since it allows the proper development of vegetation and does not limit the removal of pollutants compared to PRS which is a more common and more expensive substrate than PET because the latter is reusable in CWs.

Regarding the growth of the vegetation, it was observed that there was a better and greater growth in plants with a lower planting density, as well as a greater production of tillers. Studies on CWs with a greater effective area are necessary to obtain a clearer picture of the landscape and behavior of tropical ornamental plants in relation to their planting density in these biosystems. Plant density shows a positive effect on the elimination of pollutants, especially on COD and ammonia, however, in cells where there were more plants with a smaller planting distance, the development of new plant shoots was limited. In this sense, it is recommended that the appropriate distance for this type of polyculture plants be 40 to 50 cm in expansion in real-scale systems, in order to take advantage of the harvesting of species in these and allow species of greater foliage such as *Alpinia purpurata* and *Hedychium coronarium* which develop properly and can produce flowers in CWs.

## Figures and Tables

**Table 1:** Water quality parameters in influent and enfluent in wetlands.

Wetlands Plants in Different Substrates									
Parameters		Low Density PET	Medium Density PET	High Density PET	Low Density PRS	Medium Density PRS	High Density PSR	Control PET	Control PRS
Water temperature (°C)									
	Influent	26.45 ± 0.29							
	Effluent	24.62 ± 0.22	25.13 ± 0.20	24.83 ± 0.21	24.92 ± 0.21	25.105 ± 0.22	25.01 ± 0.23	24.88 ± 0.27	24.70 ± 0.24
pH									
	Influent	6.80 ± 0.47							
	Effluent	7.72 ± 0.43	7.70 ± 0.37	7.65 ± 0.41	7.69 ± 0.35	7.60 ± 0.42	7.63 ± 0.36	7.89 ± 0.32	7.76 ± 0.38
EC	(µS/cm)								
	Influent	1601.33 ± 291.15							
	Effluent	1116.63 ± 41.39	1128.71 ± 38.66	1028.23 ± 47.33	1079.62 ± 45.18	1014.46 ± 40.32	1190.70 ± 178.96	1041.62 ± 58.85	1064.25 ± 56.67
DO (mg/L)									
	Influent	1.7 ± 0.42							
	Effluent	3.6 ± 0.51	3.9 ± 0.26	6.7 ± 0.13	3.4 ± 0.60	4.1 ± 0.34	5.8 ± 0.72	2.4 ± 0.54	2.1 ± 0.21
TDS (mg/L)									
	Influent	670.96 ± 14.79							
	Effluent	529.08 ± 15.13	507.77 ± 16.39	505.11 ± 19.59	480.61 ± 18.66	496.01 ± 20.23	458.71 ± 18.13	585.01 ± 13.89	595.63 ± 11.75

Values are given as the average ± standard error (n = 48); different letters indicate significant differences between the columns at the 95% significance level. PSR, Porous stone river; PET, polyethylene terephthalate.
